# Mathematics intelligent tutoring system for learning multiplication and division of fractions based on diagnostic teaching

**DOI:** 10.1007/s10639-022-11553-z

**Published:** 2023-01-10

**Authors:** Shu-Chuan Shih, Chih-Chia Chang, Bor-Chen Kuo, Yu-Han Huang

**Affiliations:** 1 Graduate Institute of Educational Information and Measurement, National Taichung University of Education, No.140, Minsheng Rd., West Dist., Taichung City, 40306 Taiwan Republic of China; 2 National Taichung University of Education, Taichung, Taiwan

**Keywords:** Adaptive instruction, Diagnostic teaching, Dialogue-based, Mathematics intelligent tutoring system, Multiplication and division of fractions

## Abstract

A one-on-one dialogue-based mathematics intelligent tutoring system (ITS) for learning multiplication and division of fractions was developed and evaluated in this study. This system could identify students’ error types and misconceptions in real-time by using a block-based matching method. The adaptive dialogue-based instruction was supported by a response-driven tutoring model, which was constructed based on the diagnostic teaching methodology. Instructional strategies including provoking cognitive conflict, problem simplification and representational teaching were used in the tutoring model of the system. Effectiveness of the math ITS in remedial instruction was evaluated through a quasi-experimental study. The participants of the study were 66 sixth graders chosen from central Taiwan. They were divided into an experimental group of 35 and a control group of 31. One week after the pretest, the experimental group received 2-h one-on-one instruction via the math ITS, while the control group took a 2-h conventional teacher instruction with the same teaching content in the classroom. All participants took a post-test within 2 days after the remedial instruction. The results showed that the experimental group using the math ITS significantly outperformed the control group. Further analysis indicated that the math ITS had a significant effect on the lesser-performing group (the lower 75% in the pretest score). In addition, a usability and user experience survey showed that students were willing and likely to learn mathematics using the dialogue-based math ITS.

In recent year, the development of intelligent tutoring systems (ITSs) has drawn researchers’ attention. Especially in the past two years, amid the COVID-19 pandemic, because schools were suspended, online learning systems played an important role. An ITS is a computer-assisted tutoring program created to imitate human expert instruction, and it can be distinguished from traditional computer-aided instruction (CAI) in that it delivers instruction in detailed steps through interaction with learners. The ITS tracks students’ learning behavior by analyzing their responses at each step, and it adaptively offers learners feedback or guidance, supporting personalized instruction. After decades of development, ITSs have been implemented via a large spectrum of applications. According to a review on published ITS research during 2014–2018 (Soofi & Ahmed, 2019), mathematics applications was the second-largest field, demonstrating the growth and importance of math ITSs. However, ITSs for mathematics learning only accounted for 15.4% (6/39) of the published researches, indicating that the development of math ITSs is in its infancy. This research endeavored to develop a dialogue-based math ITS to help sixth graders learn multiplication and division of fractions.

## Dialogue-based ITSs

One specific type of ITS is the dialogue-based ITS, which captures the effectiveness of expert human teacher–learner interactions using natural language conversations with learners. A conversation begins when the computer agent poses a question, which initiates a series of dialogue moves directed toward relevant concepts (Afzal et al., 2019). The goal of a dialogue-based ITS is to scaffold knowledge akin to expert one-on-one human tutoring. Nye et al. (2014) discussed the strength of natural language-tutoring ITSs in comparison with other types of ITSs, and they found that a dialogue-based ITS integrated with an effective pedagogical design was more effective than a conventional ITS (Chi et al., 2014). AutoTutor is a competently developed dialogue-based ITS (Graesser, 2016) featuring the advantages of offering robust semantic analysis technology, an easy-to-use authoring tool, and an expandable delivery technology. Recently, ALEKS (Doignon & Falmagne, 1999), a step-based ITS for online assessment and learning of mathematics, was integrated with the AutoTutor dialogue and was found to improve significantly students’ ability to self-explain in mathematics (Nye et al., 2018). Based on the mentioned advantages, the dialogue-based math ITS of this study was developed based on AutoTutor.

## Dialogue-based ITSs for mathematics learning

Mathematics is considered an essential part of science, as mathematical analysis methods play a vital role in various disciplines, including engineering, economics, medical science, and statistics. Notwithstanding this importance, mathematics is often perceived as difficult (Acharya, 2017). Thus, the essential role of ITSs for mathematics learning has been acknowledged, and the use of ITSs to help students learn mathematics has become an important research topic (AbuEloun & Naser, 2017; Hsieh & Chen, 2019; Mokmin, 2020; Paiva et al., 2017). Referring to dialogue-based math ITSs, Kochmar et al. (2020) implemented machine learning and natural language-processing technologies in a large-scale dialogue-based ITS, named Korbit (Serban et al., 2020), and they verified the effectiveness of improving students’ learning outcomes. In the teaching program, they used a natural language-processing technique to compare students’ solutions against stored expected solutions. The recognition process was based on detecting keywords from the students’ solutions and the teaching topics of the platform related to data science, machine learning, and artificial intelligence (AI), which were designed for college students. The matching method is unsuitable for the math ITS of this study, particularly for analyzing the process of computing fractions, because of the diverse forms of fractions and orders of operations.

In the authors’ previous work (Pai et al., 2021), a Chinese-interface dialogue-based ITS was applied to remedial instruction for mathematics learning. The unit ‘multiplication and division of time expression’ was taught to fifth-grade students, and the learning performance was compared with conventional teacher instruction and material reading. The tutoring strategies were designed according to the five-step tutoring frame (Bloom, 1956) and the expectation- and misconception-tailored dialogue framework of AutoTutor (Graesser, 2016). However, in this previous work, instructional dialogues were simply designed to identify students’ mistakes or to give guidance directly. There were fewer instructional mathematics rationales introduced to the design of tutoring strategies. In summary, because of the lack of ITSs for learning fraction arithmetic, in this study, we continue improving the design of a tutoring program. The block-based matching method was used to analyze students’ inputs, and diagnostic teaching pedagogies were implemented in the tutoring program to enhance the instructional design.

## Diagnostic teaching for fraction arithmetic learning

### Diagnostic teaching

The general diagnostic teaching method includes identifying key concepts, as well as misconceptions; provoking cognitive conflict by presenting substantial open challenges; and resolving problems through intensive discussion (Bell, 1993). This approach puts students on the right track toward long-term learning by developing in them a deeper comprehension, with connections to other topics and applications. Pedagogies used in the proposed math ITS include provoking cognitive conflict, problem simplification, and representational teaching. Five strategies for provoking cognitive conflict were identified in this study, including two-to-one, reverses, one-to-many, using references, and representations (Liu, 2005), which are described as follows.

1. *Two-to-one*: when the student’s answer is incorrect, the teacher can propose another question to which this answer is correct to provoke cognitive conflict in the student. For example, when the student offers an incorrect response, such as $\frac{6}{2}\times \frac{3}{2}=\frac{9}{4}$, the teacher can propose the question $\frac{6}{4}+\frac{3}{4}= ?$ to provoke cognitive conflict.
2. *Reverses*: when the student’s answer is incorrect, the teacher can take this answer and propose a reverse question to the student. For example: when the student offers an incorrect response, such as $2\frac{1}{4}=\frac{3}{4}$, the teacher can propose the reverse question ‘please transfer $\frac{9}{4}$ to a mixed fraction’ to provoke cognitive conflict.
3. *One-to-many*: when different answers appear in class, the teacher can guide students to discuss to provoke cognitive conflict. Currently, the tutoring process designed in this study adopts a one-to-one mode, but the one-to-many tutoring strategy will be realized in a multi-agent mode in future work.
4. *Using references:* when the student’s answer is incorrect, the teacher can introduce a reference answer to the student to provoke cognitive conflict. For example: when the student offers an incorrect response, such as $2\frac{1}{4}=\frac{3}{4}$, the teacher can ask the student to compare $2\frac{1}{4}$ with the reference value 2 to provoke cognitive conflict.
5. *Representations:* it is effective to clarify students’ misconceptions through explanation and representation using diagrams or through manipulation, especially for fraction arithmetic, as there are more abstract concepts. In this study, a toolbox was integrated in the ITS to help students answer questions by manipulating bricks.

**Fig. 1:**
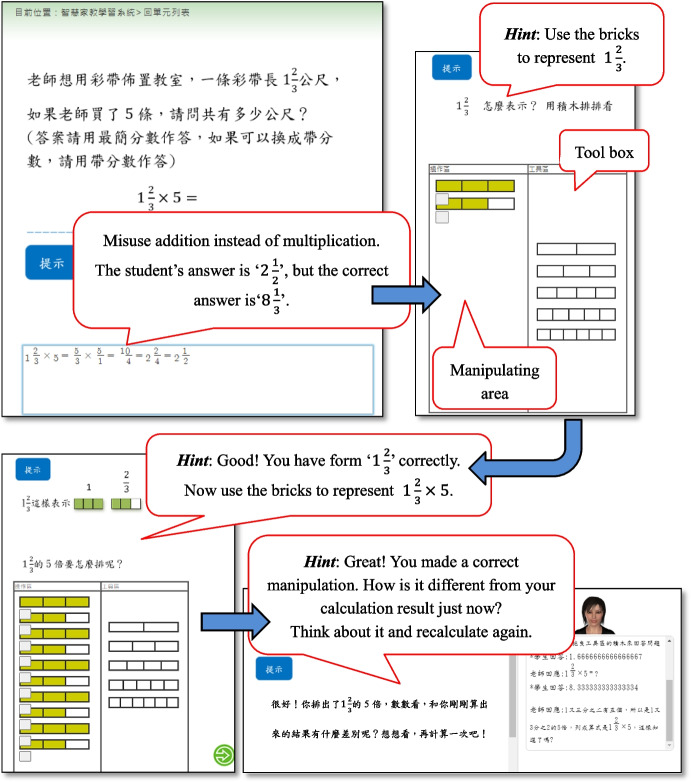
Example of representation and manipulation in the UI

During the stage of designing tutoring scripts, instructional strategies for different error types were designed according to expert suggestions. When students use the math ITS, the system can recognize the error type in their responses and provide adaptive cognitive instruction. Taking the ‘representations strategy’ as an example, as shown in Fig. 1, the main question is as follows: ‘A ribbon is $1\frac{2}{3}$ meter long, what is the total length of 5 ribbons?’ If a student has trouble computing $1\frac{2}{3}\times 5=\frac{5}{3}\times \frac{5}{1}=\frac{10}{4}=2\frac{2}{4}=2\frac{1}{2}$, due to misassigning addition instead of multiplication, the system will guide the student to represent the computing process by dragging and painting bricks step by step. By comparing the representations with the student’s response, the student will be aware of the cognitive conflict and reconsider the correct computing process.

### Difficulties of learning fraction arithmetic

In mathematics topics, fraction arithmetic is considered the most difficult for elementary students due to its multifaceted meanings, including ratios, rates, proportions, percentages, and decimals. Even for a novice teacher, teaching this topic well is an arduous task **(**Torbeyns et al., 2015**)**. Nevertheless, the skill of fraction arithmetic is crucial to later mathematics achievements **(**Brown & Quinn, 2007) and to the ability to succeed in many professions **(**Lortie-Forgues et al., 2015**)**. A major reason for the difficulties in learning fraction arithmetic is a misunderstanding of whole numbers (Gagani & Diano, 2019; Namkung & Fuchs, 2019). For instance, students view numerators and denominators as separate integers instead of one whole number, or they misapply whole number properties to compare fraction values. To help students master fraction arithmetic, in addition to the essential concepts presented in class, providing opportunities for student practice after school or for remedial instruction is crucial. Therefore, this study designed a math ITS with adaptive instruction based on diagnostic teaching pedagogies to help students learn fraction concepts individually. Because an internet-based ITS can be used in a classroom as assisting instructional equipment and can be used after class as a practice platform, the proposed math ITS will contribute to reducing the teaching load of teachers.

### Block-based matching method

In the authors’ previous work (Pai et al., 2021), the knowledge concepts of ‘multiplication and division of time expression’ relate to the skills of multiplication and division with integers. For instance, 15 min times 6 equals 90 min, which can also converted to 1 h and 30 min using division. In daily life, however, time is frequently expressed as a fraction, such as $1\frac{1}{2}$ hours. Thus, the multiplication and division of fractions represent more complicated concepts and skills (Lortie-Forgues et al., 2015). Consequently, this research aims to design a tutoring program for a dialogue-based math ITS to help students learn the concepts and skills of multiplication and division of fractions. In a math ITS, for the purpose of checking students’ answers and giving students adequate feedback, the system must analyze students’ responses. The block-matching algorithm is popular and efficient in video motion estimation, mainly due to its simplicity and superior performance (Khawase et al., 2017; Yaakob et al., 2013). Herein, the block-based matching method proposed by Yang et al.  (2011) segmented the students’ responses, which were coded in LaTeX format into blocks, and then the blocks were compared with pre-stored expectative/misconception patterns to decide whether the response is correct according to matching percentages. Because of the diversity of the computational process, the block-based matching method is especially suitable for analyzing students’ problem-solving processes, instead of detecting keywords or checking answer only.

In summary, the main contributions of this study include extending the application of the math ITS to a more complex knowledge concept and designing effective tutoring strategies by implementing diagnostic teaching rationales in the system.

### Dialogue-based math ITS for remedial instruction

Because ITSs are generally web-based, they can be accessed by students before and after school for convenient individual learning. In addition, due to the flexibility of their use, ITSs can be applied in classes to assist in teaching and learning. Shin (2021) discussed the ways teachers integrate ITSs into their lessons, stating that an ITS can be positioned as a servant or a partner with teachers. As a servant, the ITS can be used at the beginning of a lesson to assess students’ prior knowledge, and it can be used to provide students with exercise questions in class or to assign homework after class. As a partner, according to students’ performances recorded in the ITS, teachers can plan or modify instructional activities based on the content with which most students had difficulty. In addition, teachers can consider students’ learning patterns and cognitive levels, identified by the ITS, when organizing groups for cooperative learning.

It is acknowledged that ITSs can offer benefits for personalized learning (Akyuz, 2020). Thus, another feasible application of ITS in education is as a tool for remedial instruction (Pai et al., 2021), as there are several challenges for teachers arranging remedial instruction in regular classrooms. These include teachers’ workloads, non-conducive learning environments (Kasran et al., 2012), and students’ mixed ability levels (Bekiryazici, 2015), all of which make it difficult for teachers to meet each student’s needs during their scheduled teaching hours, much less to offer effective remedial instruction to underachieving students. In this study, the proposed dialogue-based math ITS was used to help sixth graders learn the skills of multiplication and division of fractions, and the pedagogical effectiveness was evaluated.

### Research questions

According to the state of ITSs in mathematics learning discussed above, this research specifically answered the following questions:

1. Does remedial instruction using the math ITS significantly improve students’ ability to solve mathematical problems involving fractions compared to traditional classroom remedial instruction?
2. Is the instruction of the math ITS effective for both high- and lesser-performing groups?
3. Is students’ feedback about the math ITS positive?

## Method

**Fig. 2:**
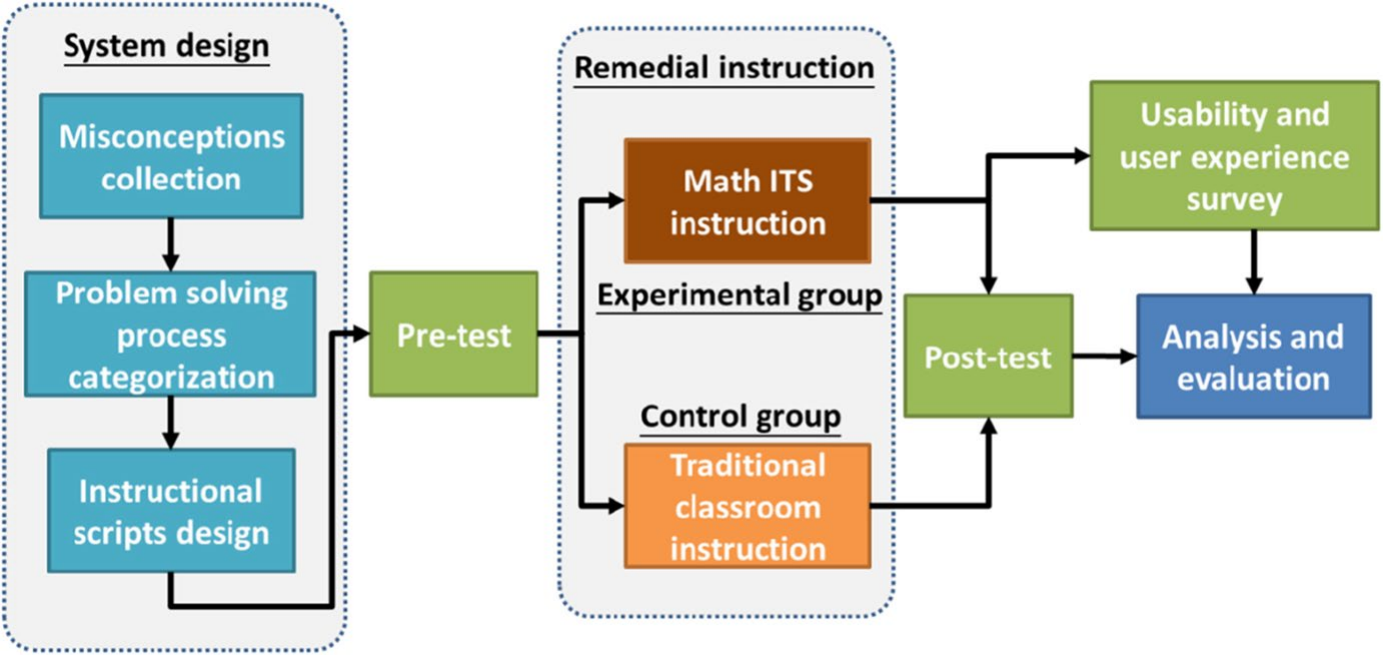
Experimental design

Figure 2 shows the quasi-experimental design of this study (Huang, 2018). Before the experiment, the tutoring corpus of the ITS was built by collecting students’ item responses from a paper-and-pencil test, categorizing expectation/misconception response patterns, and setting up tutoring scripts. Because the experiment in this study focused on remedial instruction, all students learned the tutoring materials in the ITS, and in the pilot study, students could finish these tasks within two hours, so the experimental intervention time was set at two hours. The pretest was held one week before the remedial instruction. After the pretest, the participants from the experimental group received two hours of one-on-one instruction via the math ITS, while those in the control group received two hours of traditional remedial instruction. When students in the experimental group used the ITS, they were guided by the tutor agent to finish the learning course by themselves without mathematics teachers. For both groups, the remedial instruction was held within one week after the pretest, and the teaching content was the same. Then, within two days of the remedial instruction program, all participants took a post-test in an equivalent form. The participants from the experimental group answered a questionnaire concerning the usability of and their user experience with the math ITS. All statistical analyses of the collected data were performed using SPSS version 22.

### Participants

The study enrolled 89 sixth-grade students from central Taiwan. Prior to the study, parental permission and student consent were obtained, and privacy was carefully guarded. In total, 23 participants were excluded, either because they missed the remedial teaching session or because they were absent for the post-test, leaving 66 participants, of whom 35 were randomly assigned to an experimental group and 31 to a control group.

### Architecture of the dialogue-based math ITS

**Fig. 3:**
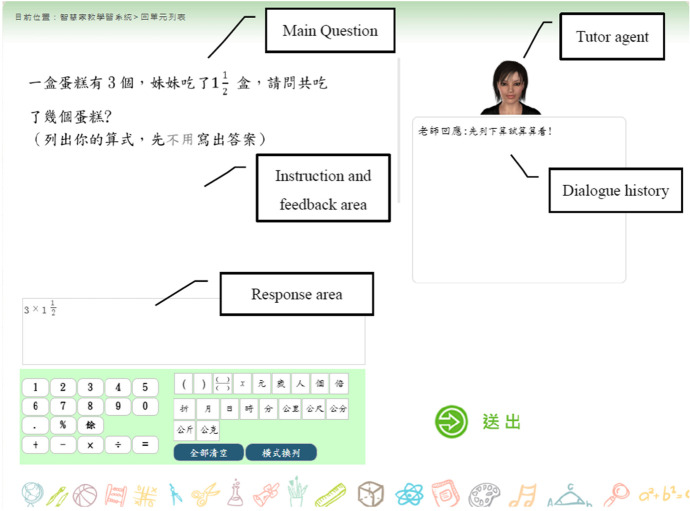
User interface of the math ITS

The user interface of the system is shown in Fig. 3. The tutoring program starts with the main question, which is displayed in the upper-left corner. Concurrently, the tutor agent, which is a talking head equipped with synthesized speech and facial expressions in the upper-right corner, gives the learner a message about the main question. The response area is in the lower-left corner; here, the learner enters an input using a keyboard and mouse. After the learner’s response has been analyzed, the agent provides encouraging feedback or guidance depending on whether the response is correct. The interaction continues until the learner has successfully solved the main question or until they fail to solve it, in which case an instructional video is presented.

The architecture and operating principle of the developed math ITS are consistent with a typical two-loop tutoring structure, which is composed of four models: the domain, tutoring, interface, and student models (Pai et al., 2021; VanLehn, 2006). The design of these models in this study is described as follows.

### Domain model

**Fig. 4:**
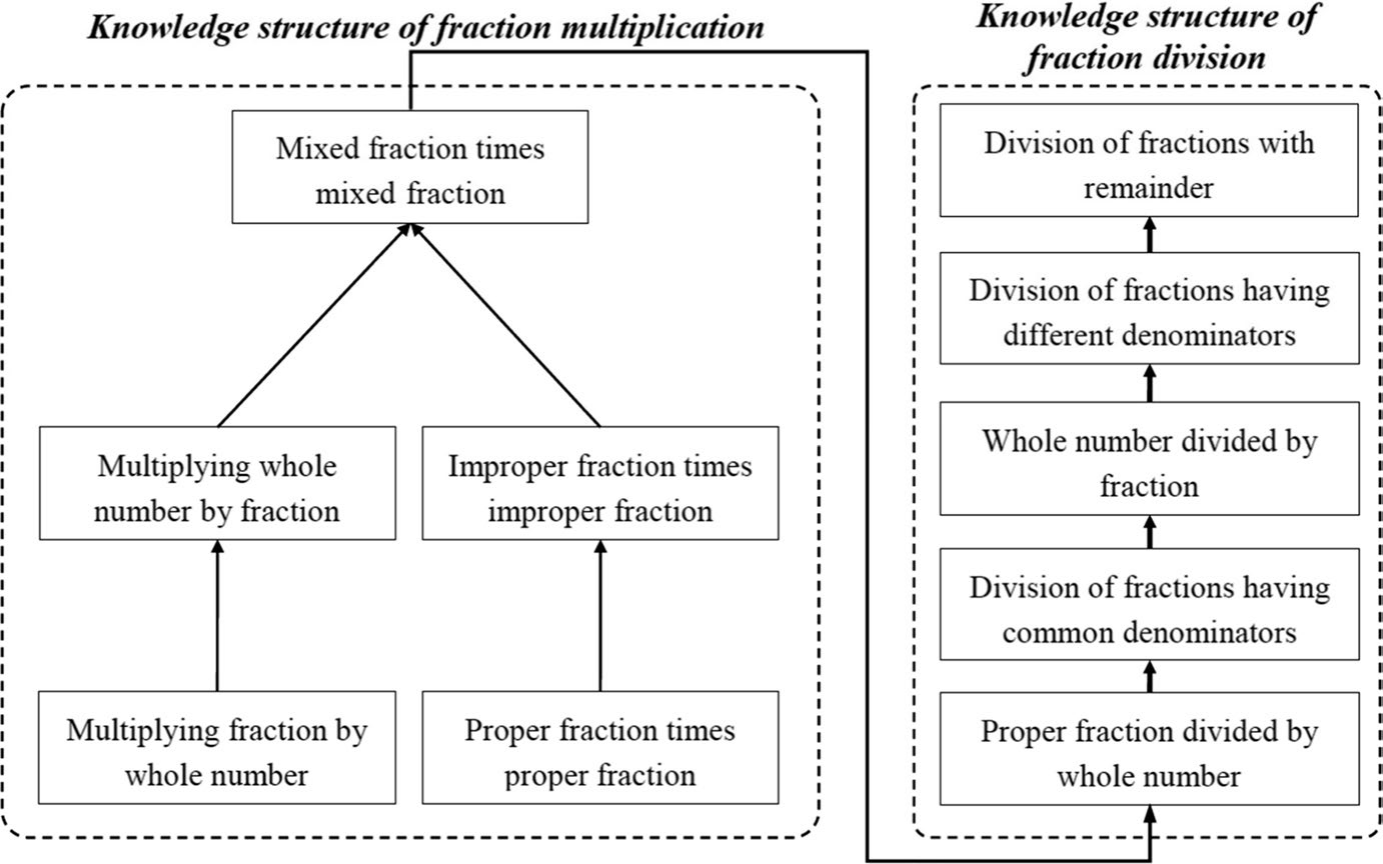
Knowledge structure of the multiplication and division of fractions

The domain model was designed in a sequential manner; that is, the sequence of assigned tasks in the learning unit was pre-determined based on the knowledge structure. Four experts and experienced mathematics teachers were invited to discuss the knowledge structure of the multiplication and division of fractions (Fig. 4). There are five skills each to learn concerning multiplication and division. Prior to learning the division of fractions, one must possess the ability to multiply fractions; for example, to calculate the quotient, one must obtain the reciprocal of the divisor and transfer the division into multiplication. In this structure, a higher node is more complex than a lower node. Thus, the main questions for learners in the tutoring system are designed and arranged according to the knowledge structure.

### Tutoring model

The tutoring model is responsible for managing the interactive tutoring procedure. The conversational scripts are stored in the tutorial corpus, and the dialogues presented are decided based on the results of the step-by-step analysis of students’ responses. An effective tutoring process relies on two important techniques: analyzing students’ responses accurately using the block-based matching method and well-designed instructional dialogues based on diagnostic teaching pedagogies. They are described as follows:

#### Block-based matching analysis

Because students’ responses to the main questions are not simply yes/no or item choices in this tutoring unit, students are asked to input the calculating procedure, in addition to the final answer. The math ITS uses the block-based matching method proposed by Yang et al. (2011), which can analyze the student’s responses, as well as their problem-solving processes. The matching method is based on a stored decision tree, which was built according to collected expectation/misconception response patterns. Possible responses to the main questions in the system was gathered from 290 sixth-grade students, who took a paper–pencil test that included 10 questions concerning the multiplication and division of fractions. After classifying these responses, the math ITS stored a set of correct answer patterns (expectations) and a set of error types (misconceptions) that occurred frequently. The decision tree of the matching procedure involves three rules:

- Rule 1. Check the status of the student’s response. If the response is blank, the tutoring procedure will return to the main question; otherwise, Rule 2 is applied.
- Rule 2. Check the correctness of the student’s formula by comparing it with the stored correct patterns in the tutorial corpus. If the formula is correct, then Rule 3 is applied; otherwise, the system will find the best matching error pattern from the stored corpus.
- Rule 3. Check the correctness of the computational process and the final answer. If they are correct, then the system records that the student has provided a correct answer; otherwise, the system finds the best-matching error pattern from the stored corpus.

For the matching analysis, the response entered by the student is first coded in the LaTeX format, and then it is segmented into several blocks by mathematical operators. Table 1 shows an example of the block-based matching of Item 3 in the system. Because the student’s response is inconsistent with the correct answer $3\times 1\frac{1}{2}=\frac{3}{1}\times \frac{3}{2}=\frac{9}{2}=4\frac{1}{2}$, the response is compared with every error type in the database, and the matching ratio is calculated. One can see in this example that the response matches error type 1 with a higher ratio, where the misconception is likely due to the misapplication of addition instead of multiplication to fractions with the same denominator. In contrast, error type 2 seems to result from the misapplication of division to fractions. According to the analyzed result, the tutoring program will lead to the corresponding instructional path.

**Table 1:** Example of block-based matching

Main question	Each box contains 3 cakes. The younger sister ate $1\frac{1}{2}$ boxes. How many cakes did the younger sister eat? (Answer the question with a mixed fraction in the simplest form.)					
The student’s response in LaTeX format and the segmented blocks	$3\times 1\frac{1}{2}=\frac{6}{2}\times \frac{3}{2}=\frac{9}{4}=2\frac{1}{4}$					
	3*1\\frac{1}{2} = \\frac{6}{2}*\\frac{3}{2} = \\frac{9}{4} = 2\\frac{1}{4}					
	$3$	$1\frac{1}{2}$	$\frac{6}{2}$	$\frac{3}{2}$	$\frac{9}{4}$	$2\frac{1}{4}$
Error type 1 with the segmented blocks in the database	$3\times 1\frac{1}{2}=\frac{6}{2}\times \frac{3}{2}=\frac{18}{2}=9$					
	$3$	$1\frac{1}{2}$	$\frac{6}{2}$	$\frac{3}{2}$	$\frac{18}{2}$	9
Error type 2 with the segmented blocks in the database	$3\times 1\frac{1}{2}=\frac{6}{2}\times \frac{2}{3}=\frac{12}{6}=2$					
	$3$	$1\frac{1}{2}$	$\frac{6}{2}$	$\frac{2}{3}$	$\frac{12}{6}$	2

#### Response-driven instruction procedures based on diagnostic teaching

**Fig. 5:**
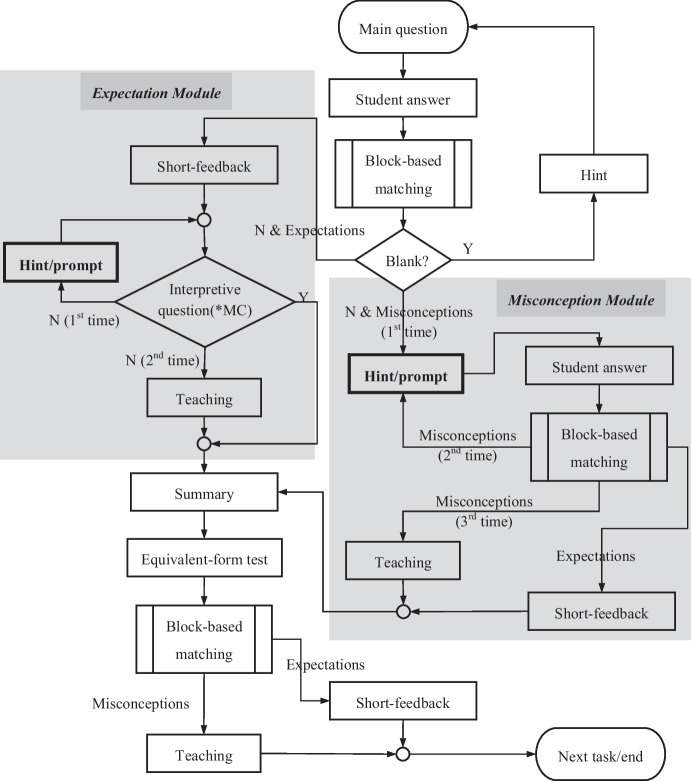
Flow chart of the response-driven instruction of the math ITS. Note: *MC = multiple choice item

The response-driven instruction procedures were designed based on the five-step tutoring frame and the expectation- and misconception-tailored dialogue framework of AutoTutor. Figure 5 shows the operating principle of the dialogue-based math ITS. The tutoring program starts by showing students the main question. Next, the subroutine of the block-based matching analysis checks the input state. If the detected response is blank, it indicates that the student pressed “Next” (leading to the next step) without typing any response. Reasons why the student may not have provided an answer include the wrong operation, misunderstanding of the main question, or unwillingness to answer. Therefore, the system gives the student a hint to remind and encourage them to answer the main question. If the input state is not blank, the student will be led to individual learning processes according to their response. Then the tutoring model is divided into two modules, one for the expectation (correct) responses and the other for the misconception (incorrect) responses. They are described separately as follows:

##### Expectation module

When the student solves the problem correctly for the first time, the system provides positive, short feedback to praise the student for answering correctly. For deep learning, the system gives the student an interpretive question. If the student answers this correctly, positive, short feedback is offered, followed by a summary of the task. If the student answers incorrectly, a hint or a prompt is provided to guide the student to think and answer again. If the student still fails to answer the interpretive question, the system provides a teaching video followed by a summary, and the tutoring for the task ends.

##### Misconception module

If the student fails to solve the problem for the first time, the system provides a hint or a prompt to help the student find the error they made and encourage them to answer the main question again. The function block ‘Hint/prompt’ in the flow chart (Fig. 5) plays the most important role in the tutoring procedure. It is responsible for delivering instructional lectures to the student. As stated, the system stores a set of frequently occurring error types, and the tutoring script’s hints or prompts were designed to correct misconceptions. Above all, the instructional dialogues were designed based on pedagogical strategies, including provoking cognitive conflict, problem simplification, and representational teaching. Table 2 shows an example of the tutoring process of Item 3 in the system, where a student was unable to solve the main question successfully. At Step 2, when the student lists a wrong formula for the first time, the tutor provides an implicit hint to remind the student to read the main question carefully and to answer it again. If the student still fails to answer the question, the tutor further assists the student with a more explicit prompt. In this case, the pedagogical methods of problem simplification and representational teaching are used. As Step 3 shows, the tutor proposes an equivalent but simpler question with an illustration to help the student discover mistakes by themself. Then, the student is encouraged to return to the main question and try again. Assume that after this instruction, the student lists a correct formula but makes some mistakes in their computation, as shown in Step 4. The tutor puts forward a question: “$1\frac{1}{2}$ boxes have fewer cakes than 1 box?” to allow the student to experience cognitive conflict. The student is expected to discover mistakes they made and try again. Finally, if the student cannot solve the main question successfully after two or three rounds of instruction, the system proposes an instructional video.

**Table 2:**
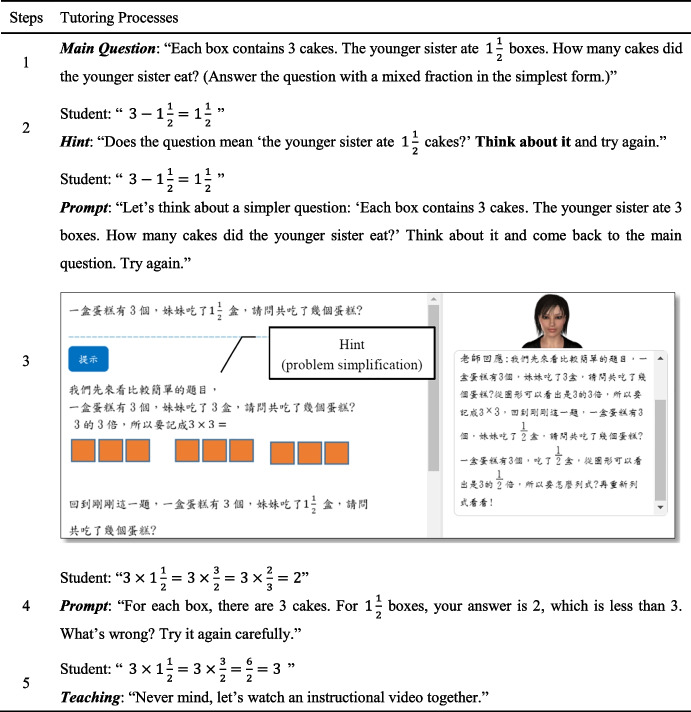
Example of diagnostic teaching

##### Equivalent-form test

Both the expectation and misconception modules end with a summary of the main question. To ensure that the student possesses the skills of this knowledge node, the student is asked to solve an equivalent-form item. This can reinforce what the student has learned. In the equivalent-form test, the analysis of the student’s response and the instruction are executed only once. Then, the system returns to the outer loop and assigns the next task to the student.

### Student model

The student model is responsible for keeping track of the student’s learning profile. It will be updated at each tutoring step. The recorded information includes the time the student spent finishing the main question, the number of tutoring loops, the student’s common error types, the tutoring path, and, finally, an evaluation of the student’s skills and competencies. The updated student model can help not only the system decide the next task, but also the teacher understand the student’s learning situation.

### Assessments

The pretest included 25 arithmetic word problems, 13 of which required students to multiply fractions and 12 to divide fractions. The post-test was in a form equivalent to that of the pretest, and Table 3 shows the items and corresponding skills tested. The pilot study showed that the discrimination of Item 16 was less than 0.2, so Item 16 was removed in the subsequent analysis. Therefore, the data analyzed included 66 participants, and the tests included 24 items with a total score of 96. Scales with Cronbach’s alpha of the pretest (α = 0.879) and the post-test (α = 0.862) indicated a high level (when α > 0.800) of reliability, and all the items in the pretest and post-test were examined by four experts and experienced math teachers to ensure the study’s content validity.

**Table 3:** Items and corresponding skills

Skill	Item	Skill	Item
S1. Multiplying a fraction by a whole number	1, 11	S6. Dividing a proper fraction by a whole number	4, 14, 22
S2. Multiplying a proper fraction by a proper fraction	7, 17	S7. Dividing fractions with common denominators	6
S3. Multiplying a whole number by a fraction	3, 13, 21	S8. Dividing a whole number by a fraction	2, 12
S4. Multiplying an improper fraction by an improper fraction	5, 15, 23	S9. Dividing fractions with different denominators	8, 18
S5. Multiplying a mixed fraction by a mixed fraction	9, 19, 25	S10. Dividing fractions with a remainder	10, 20, 24

### Usability and user experience survey

To understand users’ feelings about the math ITS, students in the experimental group were asked to complete a usability and user experience questionnaire. The questionnaire was designed as a four-point Likert scale. As show in Table 4, there were four multiple-choice items and one open-ended question in the questionnaire. Regarding the user experiences of the ITS, the first three items were designed to evaluate the acceptance of the math ITS and the fourth to compare the math ITS with traditional teacher teaching. The Cronbach’s alpha value of the questionnaire (α = 0.827) indicated a high level of reliability. The fifth question was an open-ended question used to collect students’ understanding of why they prefer the ITS or teachers’ classes when learning mathematics.

**Table 4:** Items of the usability and user experience survey

Item
1. I like using the math ITS to learn the multiplication and division of fractions
2. I would like to spend more time learning mathematics with the math ITS
3. The math ITS gives me confidence to learn mathematics
4. I feel that the interaction with math ITS is similar to that with a real teacher 5. Do you prefer the ITS or teachers’ classes for learning mathematics?

### Data analysis

To evaluate whether using the math ITS significantly improved students’ performances, a one-way analysis of covariance (ANCOVA) was conducted using the pretest score as the covariant, the remedial approaches as an independent variable, and the post-test score as a dependent variable. The descriptive statistics from the usability and user experience survey were also reported.

## Results

This study examined the pedagogical effectiveness of the dialogue-based math ITS by comparing the learning performance of the two instruction conditions. After testing whether the assumption of the homogeneity of slopes was met, at F = 2.00 (*p* > 0.05), it was reasonable to conduct a further analysis using ANCOVA. Table 5 shows the results. The experimental group significantly outperformed the control group, at F = 5.52 (*p* < 0.05, η^2^ = 0.742), and the adjusted mean was 81.57 (SE = 1.41) in the experimental group and 76.74 (SE = 1.49) in the control group, respectively. This finding reaffirmed the effectiveness of the proposed approach.

**Table 5:** ANCOVA: comprehensive results

Group	*N*	*Mean*	*SD*	Adjusted mean	Std. error	*F*	${\upeta }^{2}$
Experimental group	35	80.40	15.12	81.57	1.41	5.52*	.742
Control group	31	78.06	16.77	76.74	1.49		

*p < .05

**Fig. 6:**
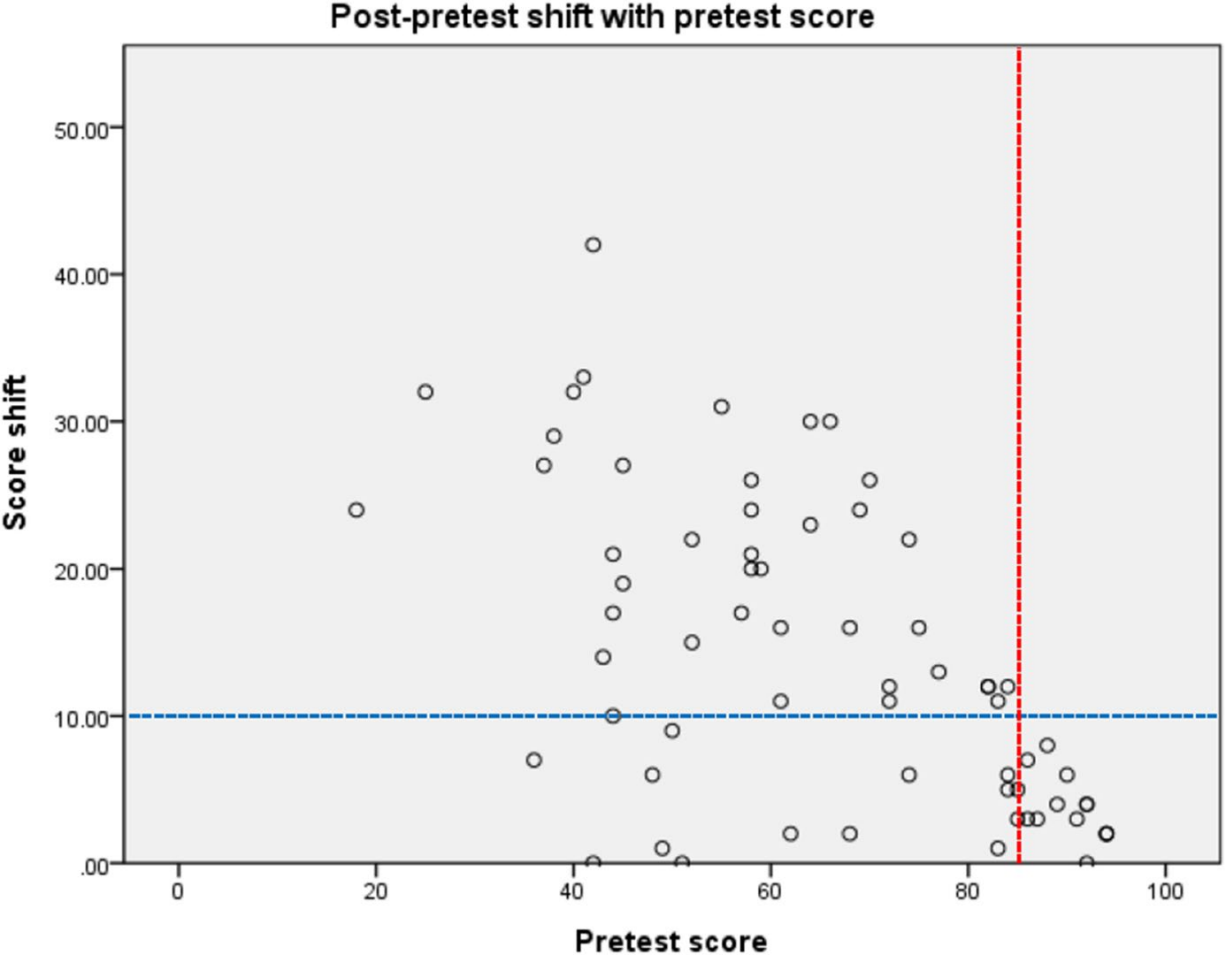
Scatter plot of the score shift versus the pretest scores

Figure 6 shows a scatter plot of the relationship between the pretest score and the score shift, which is the difference between the pre- and post-test scores. The plot shows that when the pretest score is higher than the third quartile (Q_3_ = 84) of the pretest scores, the score shift decreases rapidly. This means there is less room for improvement for students who are originally high performers. For further analysis, the data set was divided into two groups, namely, high- and lesser-performing groups, according to the third quartile (Q_3_ = 84) of the pretest scores. As Table 6 indicates, in the lesser-performing group, the experimental group significantly outperformed the control group, at F = 5.53 ( *p* < 0.05, η2 = 0.658), while in the high-performing group, there were no statistically significant differences between group means, at F = 1.15 (*p* > 0.05). According to these results, the performances of higher-performing students were not greatly impacted by the instructional method. The underachieving students, however, benefited greatly from well-designed remedial instructional programs.

**Table 6:** ANCOVA results of high- and lesser-performing groups

High-performing	*N*	*M*	*SD*	Adjusted mean	Std. error	*F*	${\upeta }^{2}$
Experimental	7	92.71	3.59	92.39	.86	1.15	.510
Control	11	93.36	2.80	93.57	.69		
Lesser-performing	*N*	*M*	*SD*	Adjusted mean	Std. error	*F*	${\upeta }^{2}$
Experimental	28	77.32	15.36	76.80	1.76	5.53*	.658
Control	20	69.65	15.17	70.38	2.08		

*p < .05

**Fig. 7:**
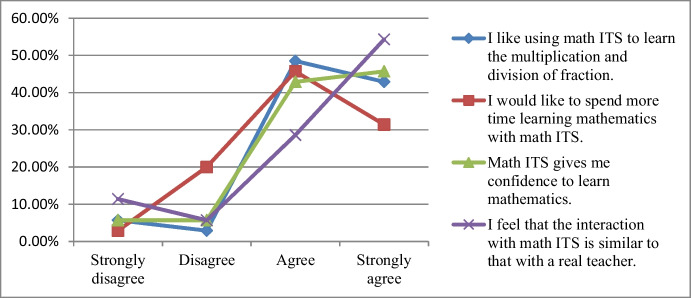
Statistic description of the feedback of using ITS survey

The results of the feedback from students in the usability and user experience survey are shown in Fig. 7, where 91.4% of students (strongly agree and agree) enjoyed learning the multiplication and division of fractions using the math ITS. In addition, 87.1% of students (strongly agree and agree) were interested in using the ITS and wanted more time to learn. Moreover, 88.6% of students (strongly agree and agree) felt more confident in learning mathematics with the ITS. These results show that the math ITS was universally accepted by students. We also asked students about the similarities in interactions between the ITS and teachers. The result showed that 82.9% of the students (strongly agree and agree) agreed that the ITS was similar to a human teacher.

## Discussion and conclusions

This study successfully developed a dialogue-based math ITS to help students learn the skills of multiplication and division of fractions. In addition, its effectiveness for remedial instruction was verified through a quasi-experimental design. Compared with extant math ITSs and the authors’ previous work, the contributions of this study are as follows: first, the application of a math ITS was extended to elementary students with a more difficult knowledge domain. All the tasks designed in this study are word problems, and to solve these problems usually requires a multi-step computing process, in addition to a correct formula. Moreover, not only can diverse representations of fractions, such as a mixed number, be represented as an improper fraction, but equivalent formulas also make the analysis of students’ responses more complex. To overcome these challenges, we gathered possible responses from 290 sixth-grade students taking a paper–pencil test in the pilot study. The system database was constructed with various expectative responses and possible error types in as much detail as possible. The correctness of students’ responses should not be checked merely by keywords or the final answer; they should be analyzed including the whole process. Because of the simplicity and efficient performance in video motion estimation, the block-based matching method was used to analyze students’ responses compared to the pre-stored responses in the database. Second, a well-designed tutorial corpus was built based on diagnostic teaching pedagogies. Although the development of an ITS benefited from AI, the essential and most important part of a computer-assisted tutoring system is the exquisite instruction program. In the authors’ previous work, no significant difference was found in pedagogical effectiveness between the math ITS and teacher instruction. In this study, the experimental results showed that the proposed math ITS significantly outperformed teacher instruction in terms of learning performance. This proved that introducing diagnostic teaching strategies into the tutoring system led to improved learning performances. Third, it was verified that the dialogue-based math ITS in this study can be used as a remedial instruction platform; moreover, the ITS can also provide students with individual practice opportunities after school. Furthermore, we think this ITS based on diagnostic teaching can also be used to teach students at the beginning of learning fractions effectively (Shih et al., 2021). Further study will continue to evaluate the effectiveness of ITS when it used at the beginning of learning fractions.

Besides, the proposed ITS was considered an accessible and useful learning tool according to students’ feedback concerning usability and the user experience. In particular, the experimental results showed that this ITS did not significantly affect the learning performance of higher-performing students, and this might be attributed to the ceiling effect (Feng et al., 2011). Moreover, a meta-analysis of the effectiveness of ITSs on mathematical learning (Steenbergen-Hu & Cooper, 2014) mentioned that the ITS was insufficient for helping low achievers. In contrast, the experimental results of this study showed that the proposed math ITS was especially effective in helping lesser-performing students, supporting the contribution of the system to remedial instruction.

In comparison to a human teacher, the math ITS has advantages, including time flexibility, offering one-on-one tutoring, step-by-step interaction with students, and learner-tailored learning profiles. However, this does not mean that instruction using ITS is better than learning from human teachers, as the ITS is usually well designed only for specific course topics. An experienced teacher can regulate the instruction content flexibly and handle any unexpected responses from students in class; this is not an available option given current AI technology. Despite these obstacles, ITSs are still helpful to teachers, as teachers face the challenge of meeting the individualized learning needs of a disparate student population with insufficient time. In contrast, with proper guidance, students can use the ITS to learn individually before and after class.

The core component of an interactive ITS is the technique of generating timely and adequate feedback to inform learners of the quality of their responses. Feedback about students’ correct answers can reinforce their performances. Concerning incorrect answers, feedback can help identify possible misconceptions and refer students to learning topics that can help them clear any confusion. The present study integrated problem-solving pedagogies based on diagnostic teaching as part of the tutoring process, which resulted in effective remedial instruction.

Despite these advantages, there are still some limitations and barriers to overcome. In the current design of the math ITS, learners must input their responses and answers using a keyboard and mouse. This is time-consuming and inconvenient, particularly for learners unfamiliar with computers, which may cause learners to become impatient. In addition, the course content taught in this study was fraction arithmetic, focused on checking and correcting computation procedures and answers. However, complex word problems can often be solved using various calculating patterns. In addition, there are always multiple steps in a calculation, and sometimes a sequence of calculations can be treated in a different order. Furthermore, when dealing with multiplication and division, there are various forms of mixed fractions. For these reasons, it was difficult to include in the system all possible patterns of learners’ responses.

## Data Availability

The authors confirm that the data supporting the findings of this study are available from the corresponding author on reasonable request.
